# Design, Synthesis, Characterization of Novel Ruthenium(II) Catalysts: Highly Efficient and Selective Hydrogenation of Cinnamaldehyde to (*E*)-3-Phenylprop-2-en-1-ol

**DOI:** 10.3390/molecules19055965

**Published:** 2014-05-09

**Authors:** Hany W. Darwish, Assem Barakat, Ayman Nafady, Mohammed Suleiman, Mousa Al-Noaimi, Belkheir Hammouti, Smaail Radi, Taibi Ben Hadda, Ahmad Abu-Obaid, Mohammad S. Mubarak, Ismail Warad

**Affiliations:** 1 Department of Pharmaceutical Chemistry, College of Pharmacy, King Saud University, P.O. Box 2457, Riyadh 11451, Saudi Arabia; E-Mail: hdarwish75@yahoo.com; 2 Department of Analytical Chemistry, Faculty of Pharmacy, Cairo University, Kasr El-Aini Street, ET 11562, Cairo, Egypt; 3 Department of Chemistry, College of Science, King Saud University, P.O. Box 2455, Riyadh 11451, Saudi Arabia; E-Mail: anafady@ksu.edu.sa; 4 Department of Chemistry, Faculty of Science, Alexandria University, P.O. Box 426, Ibrahimia, Alexandria 21321, Egypt; 5 Department of Chemistry, Science College, An-Najah National University, P.O. Box 7, Nablus 0092, Palestine; E-Mails: suleimanshtaya@najah.edu (M.S.); obaid@najah.edu (A.A.-O.); 6 Department of Chemistry, Hashemite University, Zarqa 13115, Jordan; E-Mail: manoaimi@hu.edu.jo; 7 LCAE-URAC18, Faculty of Science, University Mohammed Premier, Oujda-60000, Morocco; E-Mail: hammoutib@gmail.com; 8 LCAE, Chemistry Department, Faculty of Sciences, University Mohammed Premier, Oujda-60000, Morocco; E-Mail: radi_smaail@yahoo.fr; 9 Lab of Chemical Material, FSO, University Mohammed Premier, Oujda-60000, Morocco; E-Mail: taibi.ben.hadda@gmail.com; 10 Department of Chemistry, The University of Jordan, Amman 11942, Jordan; E-Mail: mmubarak@ju.edu.jo

**Keywords:** Ru(II) complexes, hydrogenation, diphosphine, cinnamic aldehyde, NMR

## Abstract

In this contribution, two novel supported and non-supported ruthenium(II) complexes of type [RuCl_2_(dppme)(NN)] where [dppme is H_2_C=C(CH_2_PPh_2_)_2_ and NN is N1-(3-(trimethoxysilyl)propyl)ethane-1,2-diamine] were prepared. The NN co-ligand caused release of one of the dppme ligands from [RuCl_2_(dppme)_2_] precursor to yield complex **1**. The process of substitution of dppme by NN was monitored by ^31^P{^1^H}-NMR. Taking advantage of the presence of trimethoxysilane group in the backbone of complex **1**, polysiloxane xerogel counterpart, **X1**, was prepared via sol-gel immobilization using tetraethoxysilane as cross-linker. Both complexes **1** and **X1** have been characterized via elemental analysis, CV and a number of spectroscopic techniques including FT-IR, ^1^H-, ^13^C-, and ^31^P-NMR, and mass spectrometry. Importantly, carbonyl selective hydrogenation was successfully accomplished under mild conditions using complex **1** as a homogenous catalyst and **X1** as a heterogeneous catalyst, respectively.

## 1. Introduction

Ruthenium(II) complexes chelated with mixed diphosphine/diamine ligands have received much attention because of their potential application in the field of homogeneous catalysis [1,2,3,4,5,6,7,8,9]. In 1995, Noyori discovered that complexes derived from the parent [RuCl_2_(diphosphine)_2_(diamine)_2_] can be utilized as homogeneous catalysts for the hydrogenation of α,β-unsaturated ketones [2,3,4]. In particular, these systems were found to be more effective in the chemoselective hydrogenation of carbonyl functional groups in the presence of olefins [10,11,12,13,14,15,16,17,18,19,20,21,22]. In this aspect, hydrogenations of C=C and C=O functionalities have found important applications in organic and fine chemicals synthesis [14,15,16,17,18,19,20,21,22,23,24,25,26]. The catalytic reactivity and selectivity of these complexes are due to their well-designed structural, electronic, and stereochemical features [9,10,11,12,13,14,15,16,17,18,19,20,21,22,23,24,25,26,27,28,29,30,31,32]. A high turnover frequency (TOF) can be obtained by designing suitable molecular catalysts and reaction conditions. Although there are many examples of highly efficient catalysts for olefin and ketone reduction, hydrogenation is still a challenge in terms of both the turnover frequency and the lifespan of the active catalyst [15,16,17,18,19,20,21,22,23,24].

Heterogeneous catalysts derived from diphosphine-diamine ruthenium complexes can be obtained via the introduction of T-functionalized (trialkylsilane, (RO)_3_Si-) into the diphosphine or diamine ligands. This functional group can easily promote immobilization of these complexes to a polysiloxane matrix through a sol-gel process [22,23,24,25,26,27,28,29,30,31,32,33,34]. In practice, immobilization of metal complexes enables the long term use of expensive or toxic catalysts and provides a clean and facile separation of products [23,24,25,26,27,28]. 

In previous work, we have examined the catalytic activity of mixed-ligand ruthenium(II) complexes obtained from diamine and diphosphine ligands as homogenous catalysts for the hydrogenation of unsaturated carbonyl compounds [8,9,10,11,12,13,14,15,16,17,18,19,20]. Some of these catalysts were supported on polysiloxane and examined as interphase catalysts [22,23,24,25,26,27,28]. In view of the wide interest in the activity and profile of homogeneous and heterogeneous catalysts, and as part of our ongoing research on the synthesis of supported and unsupported phosphine/diamine Ru(II) complexes for their hydrogenation activity in both homogenous and heterogeneous phases [20,21,22,23,24,25,26,27,28,29,30], we report herein on the synthesis and characterization of a novel ruthenium(II) complex [RuCl_2_(dppme)(NN)], **1**, where dppme is H_2_C=C(CH_2_PPh_2_)_2_ and NN is 3-(2-aminoethyl)aminopropyl]trimethoxysilane as well as its cross-linked xerogel **X1** through a simple sol-gel reaction using diamine functionalized with Si(OEt)_4_, as homogenous and heterogeneous catalysts for the hydrogenation of cinnamaldehyde. In this work the dppp saturated diphosphine ligand was replaced by dpme as an unsaturated diphosphine ligand, the reason behind that, being to test and compare the catalytic hydrogenation behavior of both complexes with different diphosphine ligands under identical condition. 

## 2. Results and Discussion

### 2.1. Synthetic Investigation of Ruthenium(II) Complex **1** and Xerogel **X1**

**Scheme 1:**
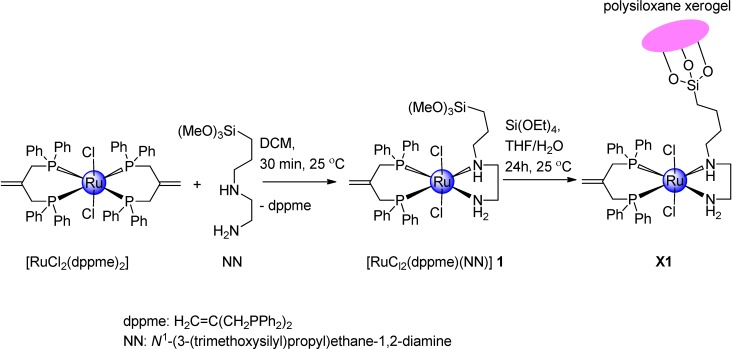
Synthesis of complex **1** and **X1**.

Reaction of [RuCl_2_(dppme)_2_] with an equivalent amount of *N*^1^-(3-(trimethoxysilyl)propyl)ethane-1,2-diamine in dichloromethane afforded complex **1** as depicted in Scheme 1. Both the change in color of the reaction mixture from brown to light yellow and ^31^P{^1^H}-NMR chemical shifts confirmed that one of the dppme ligands was exchanged equivalently by a diamine ligand. The structure of the complex **1** was confirmed by elemental analysis and by means of various spectroscopic techniques, including IR, TG/DTA ^1^H-, ^13^C{^1^H}- and ^31^P{^1^H}-NMR spectroscopy, and FAB-mass spectrometry. For this compound, the ^1^H-, ^13^C-, and ^31^P-NMR spectral features are all in excellent agreement with the suggested structure.

Complex **1** has been used to prepare the xerogel **X1** through a simple sol-gel polymerization process at room temperature in the presence of 10 equivalents of Si(OEt)_4_ as cross linker using methanol/THF/water, as shown in Scheme 1. Due to the poor solubility of **X1** in common solvents, it was subjected to solid state NMR measurements.

### 2.2. Spectral Data

#### 2.2.1. ^31^P-NMR Spectrum of Complex **1**

**Figure 1:**
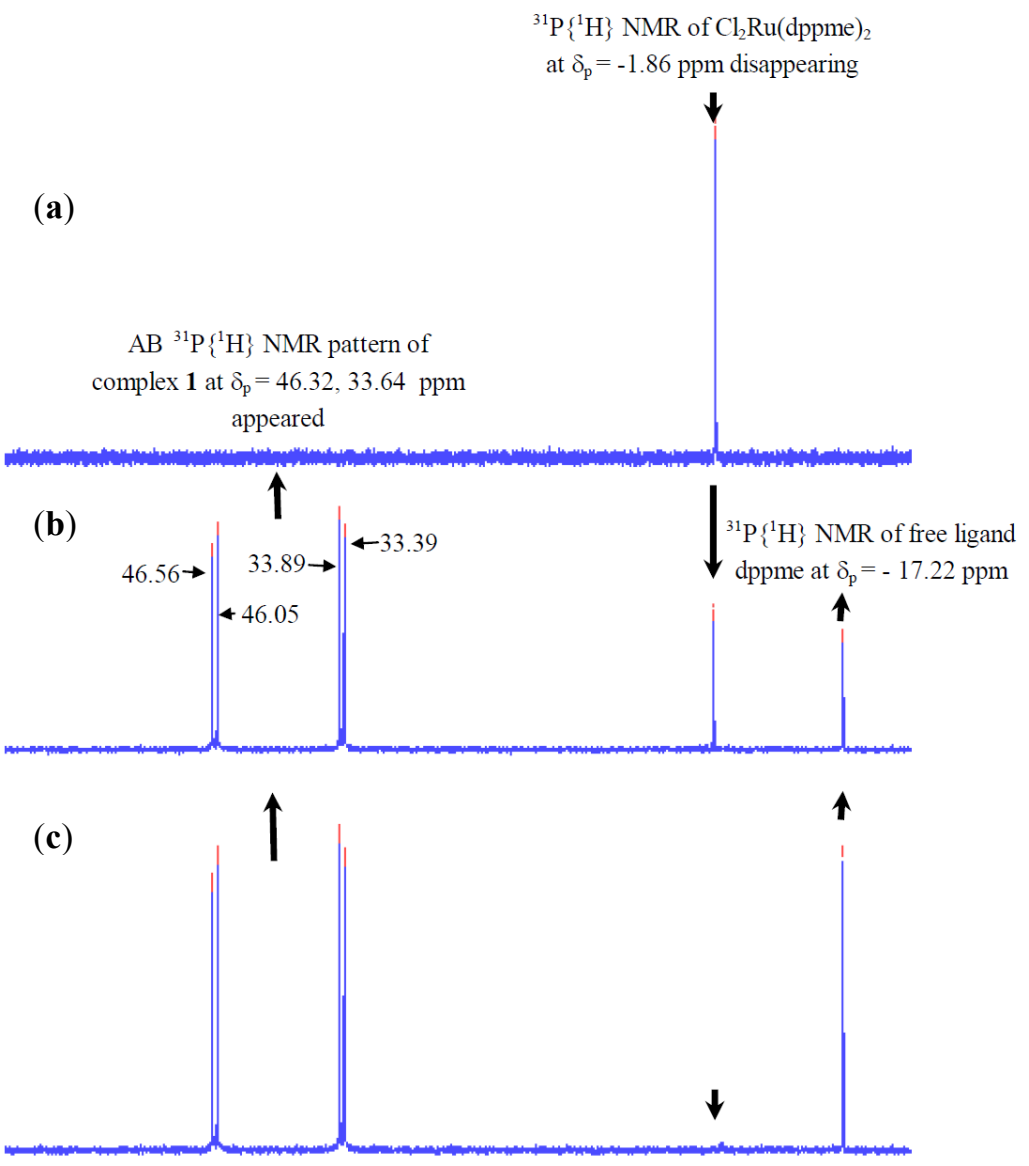
Time-dependent ^31^P{^1^H}-NMR spectrum of [RuCl_2_(dppme)_2_] at *δ*_p_ = −1.86 ppm mixed with one equivalent of [3-(2-aminoethyl)aminopropyl]trimethoxysilane co-ligand to produce complex **1** at *δ*_p_ = 46.32, 33.65 ppm (**a**) before the co-ligand addition, (**b**) 10 min. and (**c**) 30 min. after the co-ligand addition.

The ^31^P{^1^H} signals in the ^31^P-NMR spectrum of complex **1** show a splitting of the ^31^P{^1^H} signals; this is due to the asymmetric nature of diamine in *N*^1^-(3-(trimethoxysilyl)propyl)ethane-1,2-diamine co-ligand without a C2 axis which will lead to AX resonance patterns for complex **1**, as shown in Figure 1. The phosphorous chemical shifts and the ^31^P-^31^P coupling constants (*Jpp* = 35.8 Hz) suggested that the phosphine ligand was positioned *trans* to the diamine, with *trans*-dichloro atoms, to form the kinetically favored *trans*-Cl_2_Ru(II) isomer [7,8,9,10].

Formation of complex **1** was monitored by ^31^P{^1^H}-NMR spectroscopy. [RuCl_2_(dppme)_2_] complex has a ^31^P{^1^H}-NMR signal corresponding to complex Cl_2_Ru(dppme)_2_ at δ_p_ = −1.9 ppm. Addition of *N*^1^-(3-(trimethoxysilyl)propyl)ethane-1,2-diamine resulted in a fast substitution of this ligand with one molecule of dppme ligand. The substitution reaction was confirmed by a decrease in the intensity and a 48 ppm downfield shift, in addition to the appearance of two new signals, one belonging to free dppme at *δ*_p_ = −17.2 ppm and the other an AX pattern ^31^P{^1^H}signal at *δ*_p_ = 33.6, 46.3 ppm which arises from the desired complex **1** (Figure 1b). [RuCl_2_(dppme)_2_] was totally converted to complex **1** within 30 min. (Figure 1c). The ^31^P{^1^H}-NMR spectrum also confirmed the absence of any side products in this ligands substitution reaction.

#### 2.2.2. Elemental Analysis and FAB-Mass Spectrum of Complex **1**

**Figure 2:**
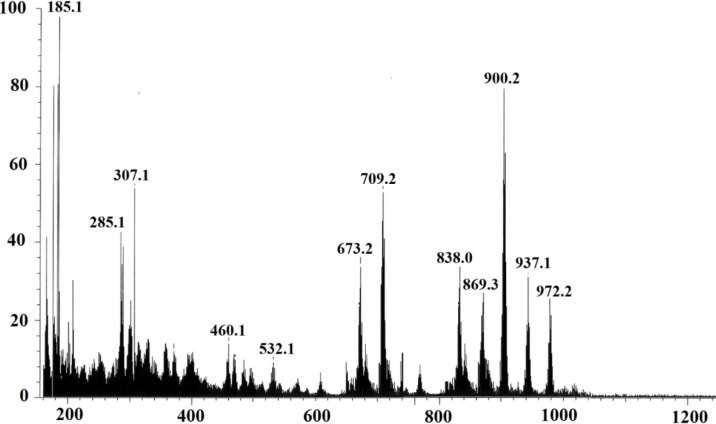
FAB-MS spectrum of complex **1**.

The elemental analysis of complex **1** is consistent with the proposed molecular formula (Calcd. for C_48_H_58_Cl_2_N_2_O_3_P_2_RuSi: C, 59.42; H, 6.12; Cl, 6.99; N, 2.68. Found: C, 59.25; H, 6.01; Cl, 7.29; N, 2.88). Additional support for the proposed structures came from mass spectral data; the FAB-MS spectrum of the complex is in good agreement with the assigned structures and showed the expected molecular ion [M^+^] *m/z* = 972.2, as suggested by its molecular formula with a 25% of the base peak intensity. Other relevant fragments that appeared in the spectrum correspond to *m/z* = 937.1 (30%) [M-Cl]^+^ and 900.2 (80%) [M-2HCl]^+^ (Figure 2).

#### 2.2.3. IR Spectrum of Complex **1**

**Figure 3:**
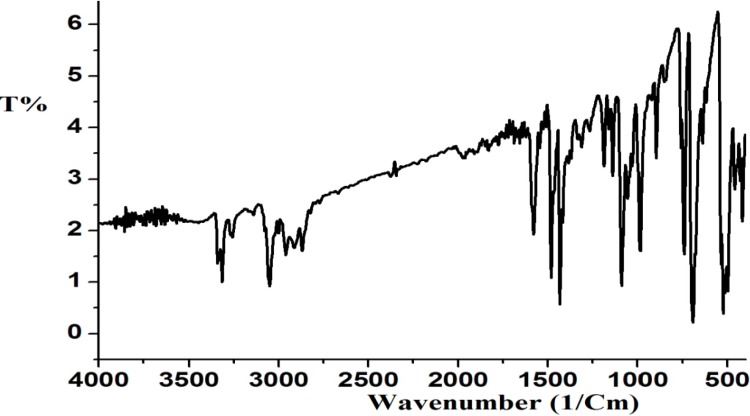
IR spectrum of complex **1**.

The IR spectrum of complex **1** shows the absorption bands of the functional groups present as displayed in Figure 3. Absorption bands in the 3,390–3,280 cm^−1^ region can be assigned to NH stretching vibrations. An absorption band observed at 3,180 cm^−1^ is attributed to the stretching vibration of the aromatic C–H bonds, while bands at 2,980–2,740 cm^−1^ are due to C-H stretching vibrations. Other characteristic bands due to other functional groups are also present in the expected regions.

#### 2.2.4. Electronic Absorption Spectral Study

**Figure 4:**
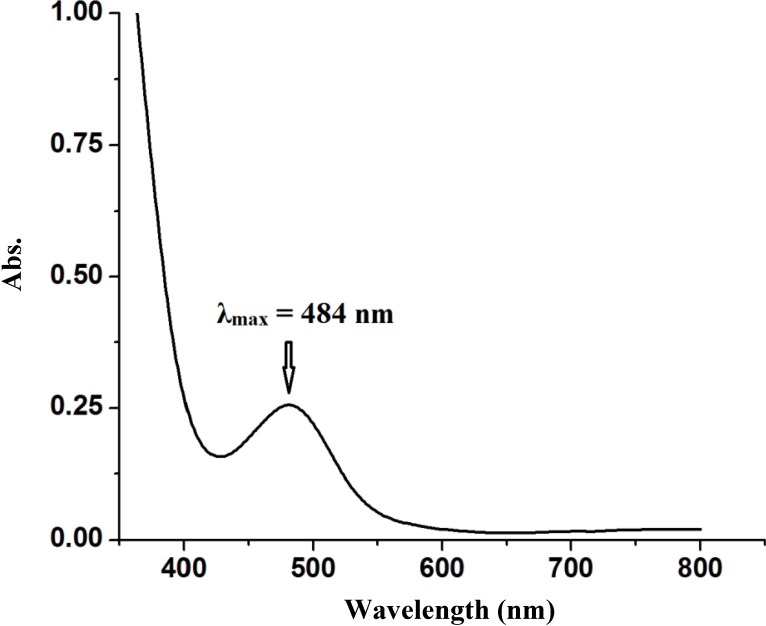
UV–Vis spectrum of the complex **1** dissolved in CH_2_Cl_2_ at RT.

The electronic absorption spectrum of complex **1** was acquired in CH_2_Cl_2_ at room temperature. Figure 4 shows the electronic absorption of the desired complex. On the basis of its intensity and position, the lowest energy transitions at 200–350 nm has been tentatively assigned to intra-ligand π-π* / n-π* transitions [7,8,9,10]. Similarly, the lowest energy transition in the visible region at 484 nm has been tentatively assigned to metal-to-ligand charge transfer transition (MLCT) [10,11,12,13,14,15,16,17,18,19,20,21,22].

#### 2.2.5. NMR Spectra of Complex **1** and Xerogel **X1**

**Figure 5:**
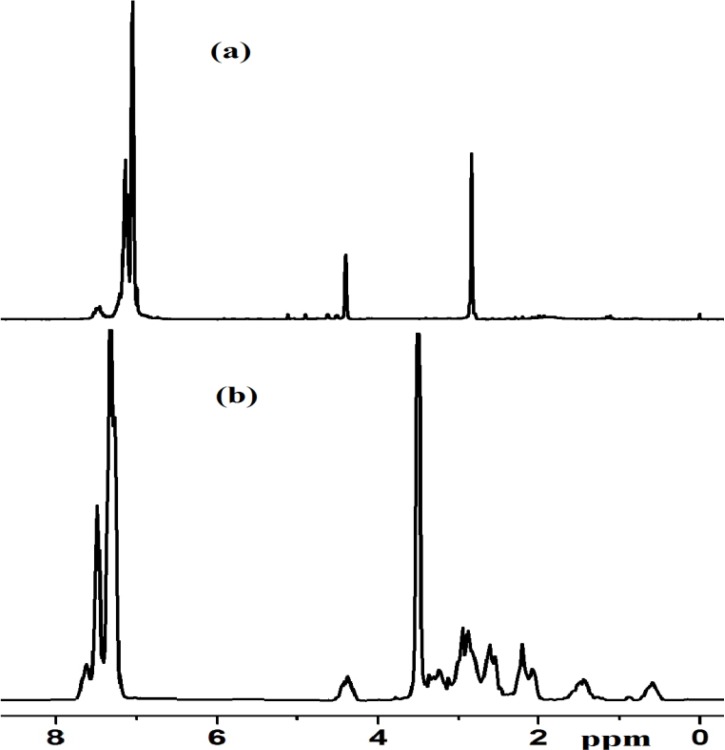
^1^H-NMR spectra of (**a**) RuCl_2_(dppme)_2_ (**b**) Complex **1** in CDCl_3_.

There is a good agreement between the ^1^H-NMR spectrum of the prepared complex **1** and its assigned structure. Displayed in Figure 5a,b are the ^1^H-NMR spectra of Cl_2_Ru(dppme) recorded in CDCl_3_ before addition of the diamine (Figure 5a) and after (Figure 5b) to prepare complex **1**. The spectrum of **1** (Figure 5b) revealed signals of aromatic and aliphatic protons of dppme and [3-(2-aminoethyl)aminopropyl]trimethoxysilane ligands that appear as complex multiplets in the region~6.0–8.0 and 0.5–4.5 ppm, respectively. Integration of ^1^H signals confirms that the dppme to diamine ratio is in agreement with the structural composition of **1**. 

**Figure 6:**
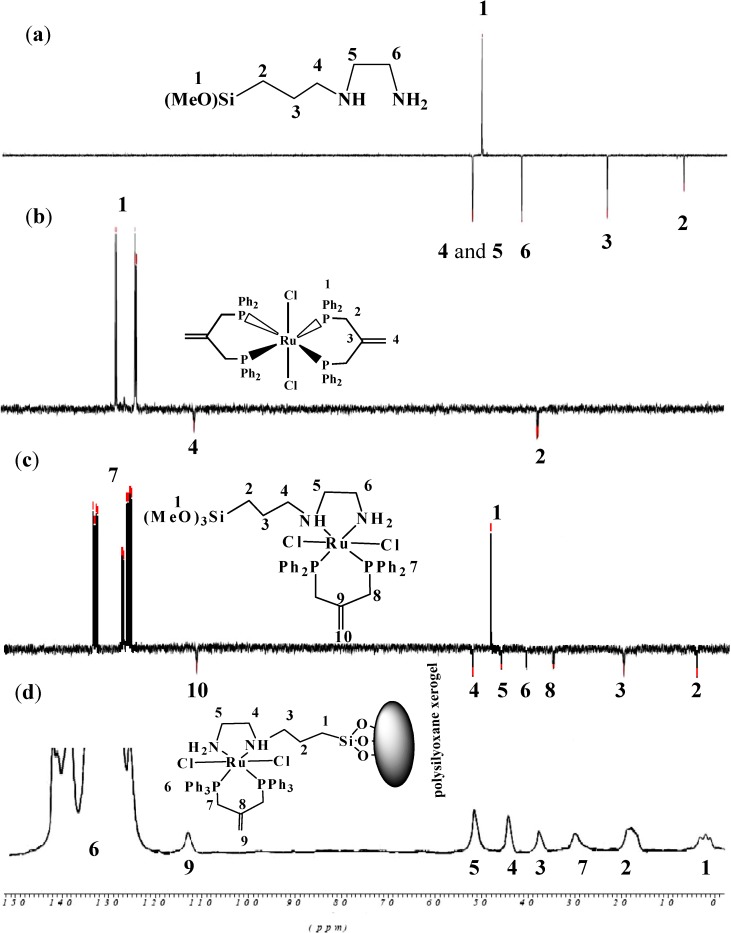
Dept 135^13^C-NMR: (**a**) free [3-(2-aminoethyl)aminopropyl]trimethoxysilane, (**b**) [RuCl_2_(dppme)_2_], (**c**) complex **1**, (**d**) solid state ^13^C-CP-MAS-NMR **X1** xerogel.

In the ^13^C-NMR spectra for complex **1** and **X1** recorded in CDCl_3_, signals associated with the different types of carbons in the dppme ligand as well as [3-(2-aminoethyl)aminopropyl]trimethoxysilane diamine co-ligand were observed. Their assignment was achieved by free ligand ^13^C-NMR and 135 DEPT studies; DEPT experiments were employed to differentiate secondary and quaternary carbons from primary and tertiary carbons. Several sets of aliphatic and aromatic carbons related to the phosphine and diamine were assigned to their positions. The ^13^C-NMR spectra of the free [3-(2-aminoethyl)aminopropyl]-trimethoxysilane, Cl_2_Ru(dppme)_2 _complex, complex **1**; reaction mixture of Cl_2_Ru(dppme)2 and [3-(2-aminoethyl)aminopropyl]trimethoxysilane] ligand and solid state ^13^C-CP-MAS-NMR of xerogel **X1** are shown in Figure 6.

Examination of the ^13^C-CP-MAS-NMR spectrum of the modified solids along with the solution phase spectrum of the corresponding molecular precursor led to the conclusion that the organic fragments in complex **1** and xerogel **X1** remained intact during the grafting and subsequent workup without noticeable decomposition (Figure 5). The absence of the CH_3_O signal at δ_C_ = 36.8 which belongs to (CH_3_O)_3_Si in [3-(2-aminoethyl)-aminopropyl]trimethoxysilane co-ligand after sol-gel reaction of complex **1** to form xerogel **X1** was the major difference recorded in spectra; this is in agreement with the immobilization of the desired hybrid Ru(II) complexes. In addition, the total disappearance of groups in **X1** (Figure 6b) compared with complex **1** (Figure 6a) provides good confirmation that the sol-gel process has proceeded to completion [22,23,24,25,26,27,28,29,30,31,32,33,34].

Solid-state ^29^Si-NMR provided further information about the silicon environment and the degree of functionalization [22,23,24,25,26]. In all cases, the organometallic/organic fragment of the precursor molecule was covalently grafted onto the solid, and the precursors were, in general, attached to the surface of the polysiloxane by multiple siloxane bridges. The presence of T^m^ sites in case of xerogel **1** and **2** in the spectral region of T^2^ at δ_Si_ = −55.8 ppm and T^3^ at δ_Si_ = −66.1 ppm was confirmed. Additionally, Q silicon sites due to Si(OEt)_4_ condensation agent were also recorded to Q^3^ at δ_Si_ = −101.2 and Q^4^ at δ_Si_ = −109.5 ppm silicon sites of the silica framework [22,23,24,25,26,32,33,34]. 

#### 2.2.6. Thermal Studies

**Figure 7:**
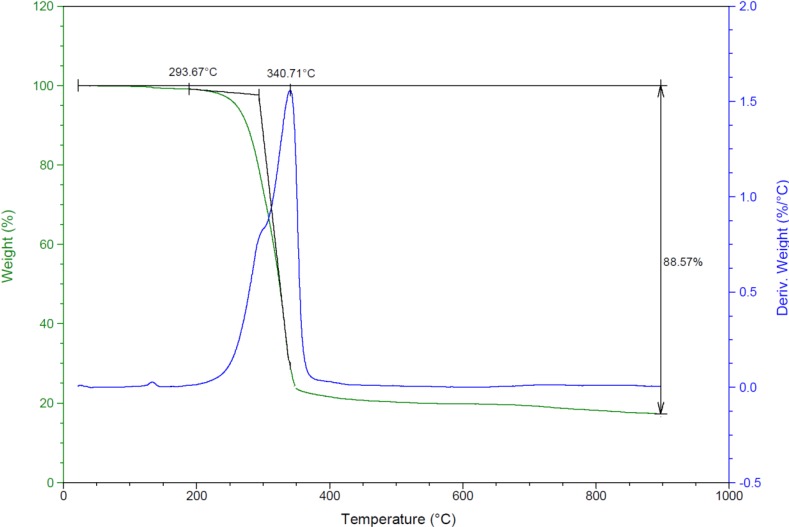
TG and DTA thermal curves of complex **1**.

A typical thermal TG/DTA curve of complex **1** is given in Figure 7. The thermal decomposition study of the complex was investigated in the 25–900 °C temperature range under open atmosphere at a heating rate of 10 °C/min. There is no weight loss in the range 25–290 °C which indicates the absence of coordinated or uncoordinated water molecules. As Figure 7 reveals, the complex undergoes a one-step decomposition with 88% weight loss due to the loss of coordinated chlorides, diamine, dppme ligands from the complex between 293 and 385 °C with an exothermic DTA peak at 340.7 °C. The final residue remaining after the complex was heated to 900 °C was analyzed by IR spectroscopy and identified as ruthenium oxide. 

#### 2.2.7. Electrochemistry

**Figure 8:**
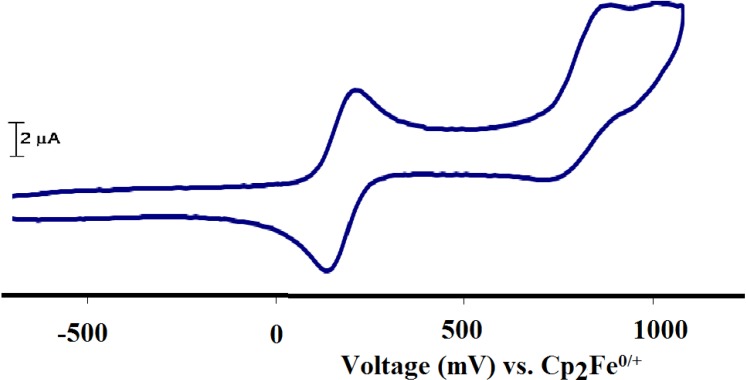
Cyclic voltammograms of complex **1** Data reported in mV *vs.* Cp_2_Fe^0/+^ (Pt-disk electrode with A = 0.0064 cm^2^, tetra-*n*-butylammonium hexafluorophosphate TBAHF, 0.1 M, CH_2_Cl_2_ at 25 °C, scan rate = 0.2 V/s).

The electron-transfer behavior of the complex **1** was examined before sol-gel by cyclic voltammetry and the corresponding results are represented in Figure 8. One-electron oxidation reversible wave (∆E = 100 mV) was observed around 0.0–0.20 V *vs.* Cp_2_Fe^0/+^ which was assigned to Ru(II/III) oxidation reduction couple reaction. The half-wave potential, E_½_~0.15 v, was calculated from the average of the anodic and cathodic wave’s potential. This is in agreement with the observed for the trans-[RuCl_2_(dpme)(diamine)] species [7,8,9].

### 2.3. Complexes **1** and **X1** as Catalysts in the Hydrogenation of Cinnamaldehyde

[RuCl_2_(diphosphine)(diamine)] has been previously used as a hydrogenation catalyst under a H_2_ atmosphere using a strong base as co-catalyst and 2-propanol as solvent for the hydrogenation of α,β-unsaturated ketones and aldehydes [2,3,4,5,6]. In this investigation, the catalytic activity and selectivity of the newly prepared complexes **1** and **X1** are studied and compared with our previous complexes. Results of the hydrogenation process using complex **1** [RuCl_2_(dppme)NN] and its polysiloxane xerogel **X1** counterpart were compared with those obtained using the complex **1**' [RuCl_2_(dppp)NN] and its polysiloxane xerogel counterpart **X1**' [22] under the same hydrogenation conditions, listed in Table 1.

**Table 1:** Hydrogenation of cinnamaldehyde with different catalysts.

Trial	Catalyst ^a^	Co-catalyst	Conversion (%) ^a^	Selectivity (%) ^b^	TOF ^c^
1	**1**	*t-*BuOK	>99	>99	1380
2	***1'***	*t-*BuOK	>99	>99	1160
3	**1**	KOH	>99	>99	1460
4	***1'***	KOH	>99	>99	1210
5	**1**, ***1'***, **X1'**, ***X1'***	K_2_CO_3_	0 ^d^	-	-
6	**X1**	*t-*BuOK	95 ^d^	93	
7	***X1***	*t-*BuOK	90 ^d^	90	
8	**X1**	KOH	94 ^d^	92	
9	***X1***	KOH	88 ^d^	92	

^a^ *Reaction conditions*: 35 °C, 2 bar of hydrogen pressure, 50 mL of 2-propanol [Ru/Co-catalysts (KOH, ^t^BuOK and K_2_CO_3_)/Cinnamaldehyde] [1:10:1000], the reaction was conducted for one hour; ^b^ yield and selectivity were determined by GC; ^c^ Turnover frequency: mole of product per mole of catalyst per hour, h^−1^; ^d^ the reaction was conducted for 12 h.

**Scheme 2:**
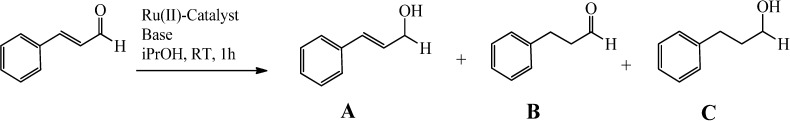
Different hydrogenation possibilities of cinnamaldehydes: Selective carbonyl group hydrogenation to produce A, selective C=C hydrogenation to produce B, full hydrogenation path with no selectivity to produce C.

Using cinnamaldehyde as model substrate, three different regioselective hydrogenation products are expected, as shown in Scheme 2. Selective hydrogenation of the carbonyl group affords the corresponding unsaturated alcohol A. Other possible hydrogenation routes (B and C) are undesired. The hydrogenation reactions using complex **1** and xerogel **X1** as catalysts were carried out under identical experimental conditions.

Taken together, the combined results clearly indicate that the newly prepared catalysts were only effective in the presence of excess of hydrogen in 2-propanol as solvent and a strong basic co-catalyst such as KOH and *tert*-BuOK. Without a strong base or even with a weak base such as K_2_CO_3_, no hydrogenation reaction was observed.

Thus, complexes **1** and **1'** are highly active under the abovementioned conditions and resulted in 99% conversion (TOF higher than 1,000) in addition to selective hydrogenation of the C=O group while keeping the C=C intact. Similarly, the xerogels **X1** and **X1'** displayed high conversion ratios and selectivity ~90% in the C=O selective hydrogenation of cinnamaldehyde using strong basic conditions. Decrease in the activity and selectivity were observed upon comparing the homogenous complexes with the heterogeneous xerogels precursors under identical condition. Moreover, complexes **1** and **X1** were highly active under identical experimental conditions compared to their corresponding complexes with dppp complex **1**' and **X1'** [RuCl_2_(dppp)(NN)] [22,23,24,25,26]. The presence of double bond in the diphosphine backbone increases the hydrogenation activity due to an increase in the electrophilicity of the ruthenium metal center [7,8,9,10].

## 3. Experimental

### 3.1. Materials and Instrumentation

Unless otherwise stated, all reactions were carried out in an inert (argon) atmosphere using standard high vacuum and Schlenk-line techniques. CH_2_Cl_2_, *n*-hexane, and Et_2_O were distilled from CaH_2_, LiAlH_4_, and from sodium/benzophenone, respectively, prior to use. Dppme, [RuCl_2_(dppme)_2_] were prepared according to literature methods [6]. [3-(2-Aminoethyl)aminopropyl]trimethoxysilane and tetraethoxysilane were purchased from Acros, (Geel, Belgium) and were used as received. Elemental analyses were carried out on an Elementar Vario EL analyzer. High-resolution liquid ^1^H-, ^13^C{^1^H}-, DEPT 135, and ^31^P{^1^H}-NMR spectra were recorded with a Bruker DRX 250 spectrometer at 298 K. Frequencies are as follows: ^1^H-NMR: 250.12 MHz, ^13^C{^1^H}-NMR: 62.9 MHz, and ^31^P{^1^H}-NMR 101.25 MHz. Chemical shifts in the ^1^H- and ^13^C{^1^H}-NMR spectra were measured relative to partially deuterated solvent peaks which are reported relative to TMS. ^31^P chemical shifts were measured relative to 85% H_3_PO_4_. CP/MAS solid-state NMR spectra were obtained with a Bruker DSX 200 (4.7 T) and Bruker ASX 300 (7.05 T) multinuclear spectrometers equipped with wide-bore magnets. Magic angel spinning was applied at 4 kHz (^29^Si) and 10 kHz (^13^C, ^31^P) using (4 mm ZrO_2_ rotors). Frequencies and standards: ^31^P, 81.961 MHz (4.7 T), 121.442 MHz (7.05 T) [85% H3PO4, NH4H2PO4 (δ = 0.8) as second standard]; ^13^C, 50.228 MHz (4.7 T), 75.432 MHz (7.05 T) [TMS, carbonyl resonance of glycine (δ = 176.05) as second standard]; ^29^Si, 39.73 MHz (4.7 T), 59.595 MHz (7.05 T, (Q8M8 as second standard). All samples were prepared with exclusion of molecular oxygen. IR data were obtained with the aid of a Bruker IFS 48 FT-IR spectrometer. Mass spectral data were acquired with a Finnigan TSQ70 (EI-MS, 200 °C) and a Finnigan 711A (FAB-MS, 8 kV), modified by AMD and reported as mass/charge *(m/z)*. Analyses of hydrogenation experiments were performed by via gas chromatography on a GC 6000 Vega Gas 2 (Carlo Erba Instruments) equipped with a flame-ionization detector (FID) and with a 10 m, PS 255 capillary column. Gas chromatographic peak areas were measured with the aid of a Hewlett-Packard 3390 A integrator. Helium (40 kPa) was utilized as a carrier gas.

### 3.2. Synthesis of Complex **1**

Complex **1** is prepared according to the following general procedure: a solution of [3-(2-aminoethyl)-aminopropyl]trimethoxysilane (0.10 g, 0.455 mmol, 5% excess) in dichloromethane (10 mL) was added dropwise to a stirred solution of Cl_2_Ru(dppme)_2_ (0.22 mmol) in dichloromethane (10 mL) over a 2 min period. The mixture was maintained at room temperature with stirring for *ca.* 1 h during which the color changed from brown to yellow. The volume of solution was then concentrated to about 2 mL under reduced pressure. Addition of diethyl ether (40 mL) caused the precipitation of a solid which was filtered (P4), dissolved in dichloromethane (40 mL), and concentrated again under vacuum to a volume of 5 mL. Addition of *n*-hexane (80 mL) caused the precipitation of a solid which was filtered (P4), washed several times with *n*-hexane, and dried under vacuum. Complex **1** was obtained in analytically pure form with very good yields. m.p. = 340 °C (dec.). ^1^H-NMR (CDCl_3_): δ (ppm) 0.52 (m, 2H, CH_2_Si), 1.48 (m, 2H, SiCH_2_C*H_2_*), 2.10 (br, 2H, SiCH_2_CH_2_C*H_2_*N), 2.21 (m, 2H, CH_2_N*CH*_2_CH_2_N), 2.54 (m, 2H, CH_2_N*C*H_2_C*H_2_*N), 2.84 (br, 4H, PC*H_2_*), 2.92 (s, 3H, NH_2_), 3.58 (br, 9H, CH_3_OSi), 4.21 (br, 2H, C=CH_2_) 6.90–7.90 (m, 20H, C_6_H_5_); ^31^P{^1^H}-NMR (CDCl_3_): δ (ppm) 33.6, 46.3, dd, AX pattern with *J*pp = 35.8 Hz, ^13^C{^1^H}-NMR (CDCl_3_): δ (ppm) 3.62 (s, C, CH_2_Si), 19.01 (s, SiCH_2_*C*H_2 _), 29.12 (m, 2C, PCH_2_), 40.84 (s, C, HNCH_2_*C*H_2_NH_2_), 43.32 (s, C, HN*C*H_2_CH_2_NH_2_), 46.83 (s, 3C, SiOCH_3_), 52.12 (s, C, SiCH_2_CH_2_*C*H_2_NH), 113.18 (s, C, C=CH_2_), 126.0–134.0 (m, 24C, C_6_H_5_); FAB–MS; *(m/z)*: 972.2 (M^+^); Anal. Calc. C, 59.25; H, 6.01; Cl, 7.29; N, 2.88; for C_48_H_58_Cl_2_N_2_O_3_P_2_RuSi: Found C, 59.42; H, 6.12; Cl, 6.99; N, 2.68%.

### 3.3. General Procedure for Sol–gel Processing of Xerogel **X1**

Complex **1** (0.100 mmol) and Si(OEt)_4_ (1 mmol, 10 equivalents) were mixed together in THF (5 mL). The sol–gel polymerization took place when a 1:1 v/v methanol/water mixture (2 mL) was added to the solution. After 24 h of stirring at room temperature, the precipitated gel was washed with toluene and diethyl ether (30 mL of each), and petroleum ether (20 mL). Finally the xerogel was ground and dried under vacuum for 24 h to afford, after workup, ~250 mg of xerogel **X1** as a pale yellow powder. ^31^P-CP/MAS-NMR: δ = 33.6, 46.3. ^13^C-CP/MAS NMR: δ (ppm) 2.68 (br, 1C, CH_2_Si), 20.12 (m, 1C, CH_2_CH_2_Si), 30.25 (m, 2C, PCH_2_), 49.31 (s, 1C, NH_2_CH_2_CH_2_NH), 44.44 (br, 1C, NH_2_CH_2_), 52.82 (s, 1C, NHCH_2_CH_2_CH_2_), 115.1 (br, C, C=CH_2_), 120.00–140.00 (m, 24C, C_6_H_5_); ^29^Si CP/MAS NMR: δ = −67.1 ppm (T^3^), −57.2 ppm (T^2^), −101.6 ppm (Q^3^), −109.5 ppm (Q^4^).

### 3.4. General Procedure for the Catalytic Studies

A mixture of 0.02 mmol of the respective complexes, 2.0 mmol of cinnamaldehyde, 0.20 mmol of KOH or *tert-*BuOK or K_2_CO_3_ as co-catalysts and 50 mL of 2-propanol was placed in a 100 mL Schlenk tube. The mixture was sonicated for 5 min to assure that the solids in reaction mixture were completely dissolved. The reaction mixture was vigorously stirred, degassed by two freeze-pump-thaw cycles, and then pressurized with hydrogen gas at 2 bars. The mixture was vigorously stirred at 35 °C for 1 h in case of homogenous or 12 h in heterogeneous. During the hydrogenation process, samples were taken from the reaction mixture after the gas was removed to determine the conversion percentage and hence turnover frequency (TOF). Samples were inserted into a gas chromatograph using a special glass syringe, and the various types of reaction products were compared with authentic samples.

## 4. Conclusions

In summary, two novel [3-(2-aminoethyl)aminopropyl]trimethoxysilane/dppme/ruthenium(II) complexes were prepared. Complex **1** was prepared in high yield by a ligand exchange substitution reaction. Sol-gel polymerization of complex **1** afforded xerogel **X1** due to the T-silyl functions on the diamine co-ligand backbone. The formation reaction of the desired complexes was monitored by ^31^P{^1^H}- NMR. The structure of complexes **1** and xerogel **X1** have been confirmed by elemental analyses, CV, IR, FAB-MS, TG/DTA and ^1^H-, ^13^C-, and ^31^P-NMR spectroscopy. When these complexes were tested as catalysts for the selective hydrogenation of cinnamaldehyde in both homogenous and heterogeneous phases, they revealed high degree of activity and excellent selectivity for carbonyl hydrogenation under mild conditions.
